# Extensive parapharyngeal and skull base neuroglial ectopia; a challenge for differential diagnosis and treatment: case report

**DOI:** 10.1590/S1516-31802010000500011

**Published:** 2010-09-02

**Authors:** Giulianno Molina de Melo, Gabrielle do Nascimento Holanda Gonçalves, Ricardo Antenor de Souza e Souza, Danilo Anunciatto Sguillar

**Affiliations:** I MD, MSc. Head and Neck Surgeon at Beneficência Portuguesa Hospital, São Paulo, Brazil.; II MD. Resident physician in Otorhinolaryngology, Beneficência Portuguesa Hospital, São Paulo, Brazil.; III MD. Pathologist at Beneficência Portuguesa Hospital, São Paulo, Brazil.

**Keywords:** Central nervous system neoplasms, Neuroglia, Congenital abnormalities, Choristoma, Head and neck neoplasms, Neoplasias do sistema nervoso central, Neuroglia, Anormalidades congênitas, Coristoma, Neoplasias de cabeça e pescoço

## Abstract

**CONTEXT:**

Neuroglial ectopia has been defined as a mass composed of differentiated neuroectodermal tissue isolated from the spinal canal or cranial cavity and remains rare. This lesion has to be considered in the differential diagnosis among newborn infants with classical symptoms of respiratory distress, neck mass and feeding difficulties. We present a rare case of extensive parapharyngeal and skull base neuroglial ectopia in 6-month-old girl who presented respiratory and feeding obstruction at birth.

**CASE REPORT:**

A six-month-old girl who presented upper respiratory and feeding obstruction at birth and was using tracheostomy and gastrostomy tubes was referred to our institution. Complete surgical excision of the mass consisted of a transcervical-transparotid approach with extension to the infratemporal fossa by means of a lateral transzygomatic incision, allowing preservation of all vital neurovascular structures. The anatomopathological examination showed a solid mass with nests of neural tissue, with some neurons embedded in poorly encapsulated fibrovascular stroma, without mitotic areas, and with presence of functioning choroid plexus in the immunohistochemistry assay. Neurovascular function was preserved, thus allowing postoperative decannulation and oral feeding. Despite the large size of the mass, the child has completed one year and six months of follow-up without complications or recurrence. Neuroglial ectopia needs to be considered in diagnosing airway obstruction among newborns. Surgical treatment is the best choice and should be performed on clinically stable patients. An algorithm to guide the differential diagnosis and improve the treatment was proposed.

## RESUMO

**CONTEXTO:**

Tecido neuroglial ectópico é definido como uma massa composta de tecido neuroectodermal diferenciado, isolado do canal espinhal ou da cavidade craniana e permanece raro. Esta lesão tem de fazer parte do diagnóstico diferencial em neonatos com os clássicos sintomas de obstrução respiratória, massa cervical e dificuldade para alimentação. Descreve-se um raro caso de tecido neuroglial ectópico extensor em parafaringe e base de crânio em uma menina de seis meses de idade que apresentou os sintomas de obstrução respiratória e alimentar ao nascimento.

**RELATO DE CASO:**

Menina de seis meses de idade que apresentou obstrução respiratória alta e alimentar ao nascimento, usando traqueostomia e gastrostomia, foi encaminha à instituição. A ressecção cirúrgica completa da massa utilizou um acesso transcervical-transparotídeo com extensão para fossa infratemporal pela incisão lateral transzigomática, permitindo identificação e preservação das estruturas neurovasculares vitais. O exame anatomopatológico evidenciou uma massa sólida com ninhos de tecido neuronal com alguns neurônios imersos em estroma fibrovascular, de pouca cápsula, sem áreas de mitoses e presença de plexo coroide funcionante ao exame imunoistoquímico. As funções neurovasculares foram preservadas na cirurgia, permitindo a decanulação e alimentação via oral no pós-operatório. Apesar do grande tamanho da massa, a criança completou um ano e meio de acompanhamento sem quaisquer complicações ou recorrência. O tecido neuroglial ectópico deve ser considerado no diagnóstico de obstruções de vias aéreas no neonato. O tratamento cirúrgico é o melhor e deve ser feito no paciente clinicamente estável. Um algoritmo para orientar o diagnóstico diferencial e melhorar o tratamento foi proposto.

## INTRODUCTION

Neuroglial heterotopia (ectopia) has been defined as a mass composed of differentiated neuroectodermal tissue isolated from the spinal canal or cranial cavity, representing heterotopias rather than neoplasms. It remains rare, with reports on neuroglial heterotopic tissue in the scalp, neck, palate, lips and middle ear, while there are fewer descriptions on neuroglial heterotopia in the pharynx and parapharyngeal space.^1,2^

The etiology is unclear. Several mechanisms have been proposed but none have been able to explain this abnormality satisfactorily. Differential diagnosis needs to be done in relation to cervical compressive masses such as meningoencephaloceles, teratoma, cystic hygroma, vascular malformation, deep parotid tumors, schwannomas or branchial cleft cysts. The lesion is usually found in newborn infants for whom no gestational abnormalities were observed, and it is very rare in adults.^2^

The classical symptoms are respiratory distress, neck mass and feeding difficulties. Computed tomography (CT) and magnetic resonance imaging (MRI) are useful for ruling out diagnoses of dura mater communication and cranial defects.^3^

The ideal timing for surgical excision is debatable. Almost all reports agree on early surgical excision, but some authors advocate delayed resection and even conservative treatment.^2,4^

We present a rare case of extensive parapharyngeal and skull base neuroglial heterotopia in a six-month-old girl who presented respiratory and feeding obstruction at birth in another service, from which she was referred to our institution for treatment. The literature is reviewed and the diagnostic tools, histopathology and surgical management are discussed.

## CASE REPORT

A six-month-old white girl was admitted to our service with a previous history of respiratory distress and inability to feed at birth, reported from another institution. Her condition prompted tracheostomy and gastrostomy. The mother’s prenatal course had been unremarkable, with no previous history of trauma, infection during pregnancy or other abnormalities.

**Figure 1.**
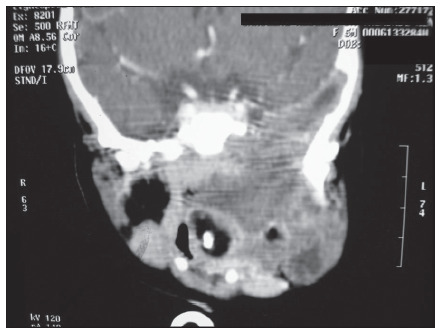
Neuroglial heterotopia shown by magnetic resonance imaging.

Neonatal CT and MRI showed an extensive heterogenous mass on the left side of the skull base, without intracranial connection. The mass extended to the ipsilateral parapharyngeal space and downward to the submandibular triangle and anterior cervical region. It dislodged the left mandibular ramus anteriorly and crossed the midline at the level of the nasopharyngeal and retromaxillary space (Figure 1).

At admission, the child presented deteriorated general status, moderate to severe malnourishment and bilateral bronchopneumonia. Because of her clinical condition, surgical treatment was postponed to allow the clinical data to become more favorable through improvements in nutritional status, respiratory assistance and clinical vital signs, in the hospital. Over this period, the mass did not show any enlargement, and there were no abnormalities in the patient’s neurological development. After three months, surgical treatment was indicated. Because there were no suitable facilities for surgical treatment at the locality where the patient lived, she was transferred to our center to be operated. After three months of enteral nutrition therapy, her nutritional status allowed the intervention.

Complete surgical excision was achieved using a transcervical-transparotid approach with extension to the infratemporal fossa, by means of a lateral transzygomatic incision.^4,5^ This enabled early identification of the facial nerve and preservation of all of the vital neurovascular structures and as much as possible of the naso-oropharyngeal mucosa. The neuroglial heterotopia presented strong adherence to the adjacent soft tissue, with areas of solid and cystic components. The dura remained intact and there were no intraoperative complications. The postoperative period was uneventful, with normal function of the vagus, hypoglossal, glossopharyngeal and accessory nerves. The tracheostomy remained necessary during the first month, until nasofibroscopy showed complete healing of the mucosa. The child received speech therapy for two months. Feeding was accomplished through the gastrostomy for two months, which was then closed after development of secure oral intake. The child left the hospital after the second month without any complications and has remained free from recurrences for one and a half year of follow-up with MRI investigations.

**Figure 2.**
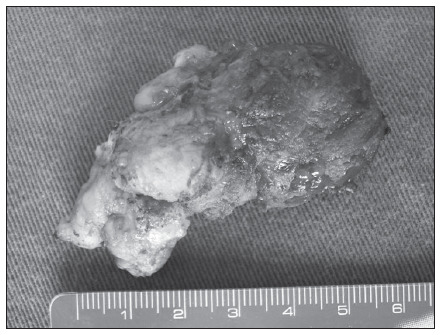
Macroscopic view of the neuroglial heterotopia.

**Figure 3.**
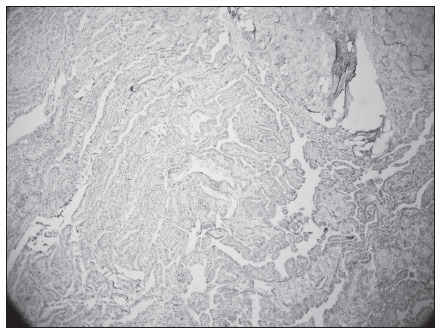
Microscopic view with immunohistochemical assay on neuroglial heterotopias (100 x, imunohistochemistry with transthyretin TTR).

The pathological evaluation on the neuroglial heterotopia showed that macroscopically, it consisted of a solid, brownish, avascular mass that adhered to surrounding tissues, with some cystic areas inside it (Figure 2). Microscopy showed nests of neural tissue with some neurons embedded within poorly encapsulated fibrovascular stroma, without any mitotic areas, and with the presence of functioning choroid plexus in the immunohistochemistry assay (Figure 3). The entire specimen was composed only of ectodermal elements.

## DISCUSSION

Since the first report by Reid in 1852, several mechanisms have been proposed to explain the origin of neuroglial heterotopia. It may derive from encephalocele;^1,2^ it may be due to separation of extracranial embryonic neural tissue through changes in the timing of cranial closure; or it may derive from isolated remains of pluripotent neuroectodermal cells that differentiated into mature neural tissue. However, other authors have postulated that it could result from aspiration of brain tissue fragments from amniotic fluid. Pathologically, neuroglial heterotopia is composed of a variety of elements of the central nervous system (ectodermal elements alone), such as astrocytes, oligodendroglia and neurons, ependyma, retinal components and choroid plexus. The cells do not present any mitoses, with fibrovascular stroma that are poorly encapsulated and adhere to surrounding tissues. The choroid plexus with liquor production is the most interesting feature in neuroglial heterotopia, demonstrated by positive findings of polyclonal antibody against transthyretin (TTR) in immunohistochemical assays.^6^

CT and MRI show a heterogenous mass surrounding by normal tissue. They make it possible to evaluate the intracranial extent of the mass and easily diagnose multi or unilocular cysts.^3^

The formal surgical indication occurs when the neuroglial heterotopia expands, thereby compromising the airway or digestive tract. The tendency is to operate as soon as possible, although some authors advocate postponement until the clinical status improves. In some cases, as in ours, surgical resection of the neuroglial heterotopia can be delayed until better conditions are attained, because of the patient’s worsened nutritional and clinical status consequent to late diagnosis.

The surgical approach consisted of transcervical-transparotid access with extension to the infratemporal fossa by means of a lateral transzygomatic incision.^4,5^ This optimal field made it possible to achieve complete resection without lesions.

The differential diagnosis includes glioma, teratoma, dermoid cyst, hemangioma, cystic hygroma, neurofibroma, branchial cyst and sarcoma. An incisional or fine-needle aspiration biopsy and immunohistochemistry assay with TTR antibodies, as part of the preoperative workup will help to determine the correct diagnosis before the surgical treatment.

To review the literature in the PubMed and Lilacs medical databases, we used the following descriptor: (Neuroglial Heterotopia Tissue) OR (Heterotopia Brain Tissue) OR (Heterotopia Neuroglia) OR (Heterotopic Neuroglial Tissue) OR Neuroglia Head and Neck OR (congenital abnormalities head neck heterotopia) OR (Choristoma head neck brain tissue). The search strategy produced a total of 751 articles from 1961 to 2009 in PubMed and seven articles in the Lilacs medical library. Among these, we considered that only 209 articles focused on the head and neck region and, after careful reading, 90 articles were excluded due to repetition. Thus, we found 119 articles consisting of case reports and case series in PubMed, while there were only three papers in the Lilacs medical database (Figure 5). The clinical data from this literature review are shown in Table 1.

**Figure 4.**
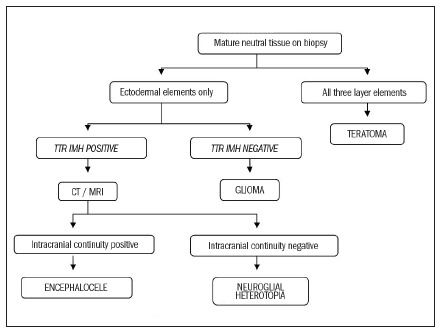
Proposed algorithm for differential diagnosis of biopsied mature neural tissue.

**Figure 5.**
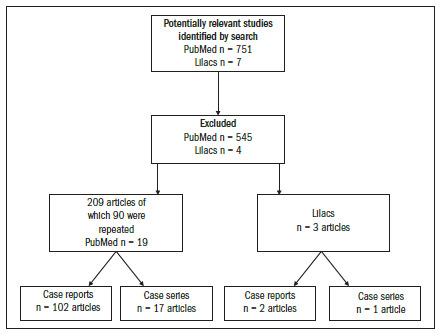
Flowcharts of search results.

**Table 1.** Literature review: neuroglial heterotopia in the head and neck region (1961-2009)

	Patients (n = 181)	
**Age**	n	%
Infant	178	98.3
Adult	3	1.7
**Neuroglial presentation**	n	%
Oral cavity	4	2.2
Tongue	16	8.8
Palate	20	11.0
Lip	2	1.1
Pharynx	7	3.8
Oropharynx	5	2.7
Nasopharynx	9	4.9
Parapharynx	21	11.6
Nasal	29	16.0
Sinus	1	0.5
Mastoid	16	8.8
Face	7	3.8
Lid	1	0.5
Scalp	15	8.2
Skin	1	0.5
Neck	5	2.7
Submandibular	3	1.6
**Treatment**	n	%
Surgical	180	99.4
Clinical	1	0.6
**Upper airway obstruction**	n	%
Yes	88	48.6
No	93	51.4
**Preoperative misdiagnosis**	n	%
Yes	180	99.4
No	1	0.6
**Recurrence**	n	%
Yes	1	0.6
No	180	99.4

Upper airway obstruction was present in 48.6% of the patients in our literature review. The initial hypothesis was correct in only 0.6 % of the cases, as in the present report.

The algorithm proposed by the authors in Figure 4 may help to elucidate the preoperative diagnosis, thereby enabling better planning of the surgical resection.

## CONCLUSIONS

Neuroglial heterotopia needs to be considered in diagnosing airway obstruction among newborns. Surgical treatment is the best choice and should be performed on clinically stable patients. Despite the large size of the neuroglial heterotopia, the present case did not show any serious neurovascular complications. The patient remains free from recurrences, after one and a half years of follow-up. An algorithm was proposed in order to aid in the preoperative differential diagnosis, thereby improving the treatment.
